# “*Seems Like There Is No Stopping Point at All Whatsoever*”: A Mixed-Methods Analysis of Public Health Workforce Perception on COVID-19 Pandemic Management and Future Needs

**DOI:** 10.3390/ijerph20146350

**Published:** 2023-07-12

**Authors:** Tony Lee, Benjamin J. Becerra, Monideepa B. Becerra

**Affiliations:** 1 Department of Health Science and Human Ecology, Center for Health Equity, California State University, San Bernardino, CA 92407, USA; 2 Department of Information and Decision Sciences, Center for Health Equity, California State University, San Bernardino, CA 92407, USA

**Keywords:** public health workforce, COVID-19, staff shortage, staff burnout, mental health

## Abstract

Objective: The COVID-19 pandemic, caused by a highly contagious novel virus called SARS-CoV-2, has led to significant global morbidity and mortality, with disproportionate burden among frontline workers. While the current empirical body of evidence highlights reported depression, burnout, moral injury, compassion fatigue, and post-traumatic stress among healthcare workers, similar assessment among the public health workforce is limited. Given work-related pressure of rapid pandemic management strategies, risk of exposure, potential fatigue, etc., understanding the caregiver burden of the public health workforce is critical. **Methods:** This study used a convergent parallel mixed-methods design. Participants were recruited using a mix of both convenience and snowball sampling. All data were collected virtually and kept anonymous. All statistical analyses were conducted using SPSS version 28, and all qualitative results were thematically analyzed using the grounded theory approach. **Results**: Among the study participants, nearly 65% reported that their personal lives were impacted due to providing COVID-19 related services. Furthermore, a majority (88%) reported poor sleep health, including low daytime wakefulness, while 24% reported serious psychological distress. Qualitative analysis demonstrated several emergent themes, with central themes indicative of the need for paradigm shift in capacity building for public health emergency preparedness that integrates caregiver support. **Conclusions:** Results highlight the importance of addressing the caregiver burden experienced by public health and related workforces during public health emergencies.

## 1. Background

In December 2019, a highly contagious novel virus, SARS-CoV-2, was discovered in China and rapidly spread throughout the world (Centers for Disease Control and Prevention [CDC], [1]), and, in March 2020, Coronavirus Disease 2019 (COVID-19) was declared a pandemic [2]. Including the global impact on mortality, COVID-19 also brought to attention the burden among those in the healthcare workforce (HCW). The HCW, which consists of physicians, nurses, emergency medical personnel, laboratory technicians, dentists, pharmacists, rehabilitation therapists, and other frontline staff, have an increased risk of COVID-19 infection due to direct exposure to and prolonged close contact with COVID-19 patients [3]. 

However, while most of the current literature has focused on the HCW, limited research, however, exists among the public health workforce (PHW). For example, Bendau et al. noted that that, during the pandemic, health professionals reported higher cases of acute symptoms related to depression, anxiety, insomnia, and even psychological distress [4]. Mittal and colleagues note that mental health providers experienced burnout, as well as poor outcomes related to both physical and mental health [5]. In fact, a query in PubMed using key words of “mental health” or “burnout” and “health professionals” and “COVID” or pandemic reveals that a majority are focused primarily on nurses and other clinicians, including mental health professionals, with limited research on the public health workforce.

The PHW, such as epidemiologists, contact tracers, case investigators, and public health educators, have been tasked with the various risk management and mitigation strategies of COVID-19, such as community outreach, contact tracing, testing, and vaccination. Further, between July and October 2020, case investigators increased by more than 50%, and contact tracers increased by about 90% [6], demonstrating a higher demand in the workforce. Despite this rapid increase, there remains limited knowledge on the magnitude of pandemic fatigue that the PHW has experienced. Given the burden of COVID-19 itself, coupled with the work-related pressure of pandemic management strategies, it is imperative to address not only the mental health status, but also stressors related to work tasks, workload, and needed resources for effective future emergency management. However, there remains a paucity of such studies among the PHW. Stone et al. [7] conducted a cross-sectional survey among health professionals from August 2020 to September 2020 to assess burnout and mental health status during the COVID-19 pandemic and noted that approximately 66% reported burnout while 46% had anxiety or depressive disorder. In a qualitative assessment of the PHW, Scales et al. [8] further reported emergent themes related to workforce environment, including team members and adaptability of workplace, career growth, constraints related to hiring and burnout, as well as politicization. Such limited empirical evidence for the PHW, despite the plethora of similar studies among other health-related workforces, highlight a need for PHW assessments to better prepare for the next public health emergency. As such, in this study, we aimed to expand the literature by employing a mixed-method approach to assess the mental health burden of the PHW, the unique experiences and barriers faced during pandemic management, and needed resources for more effective strategies for future public health emergencies. 

## 2. Methods

We utilized a convergent parallel mixed-methods [9] approach with a mix of both convenience and snowball sampling, to assess the mental health outcomes associated with the COVID-19 pandemic among the public health and the related workforce. All participants were adults over the age of 18 years employed in public health discipline in the U.S. at the time of the survey. 

In this study, we chose to utilize a mixed-methods approach for two primary reasons. While quantitative assessment can provide needed numerical data to demonstrate patterns, qualitative analyses can provide in-depth understanding of such patterns and/or reveal additional emergent themes. Often, experts describe qualitative as providing information from within and, thus, assisting in the goal of exploring new phenomena that cannot be discerned using existing theories, which is not the case for quantitative. Qualitative studies also utilize non-standardized and, thus, adaptable methods of data collection and allow for more in-depth discussions. Quantitative is deductive, while qualitative is inductive. Quantitative also relies on structured questionnaires and statistical calculations, which stands in contrast to qualitative, which uses semi-structured questions and in which analysis is more descriptive in nature [10]. A mixed-methods study can, thus, provide the benefits of both approaches. Although there remain a variety of mixed-methods [11], such as explanatory (quantitative first followed by qualitative) or exploratory (qualitative first followed by quantitative), in our study, we utilized the convergent parallel method to ensure that each approach provided a mix of outcomes without one influencing the other. 

To recruit participants, the co-investigators of the study reached out to professional networks with a recruitment email and link to complete the survey. The recruitment email also asked potential participants to share with their public health professional network. As approved by the Institutional Review Board, all participants who currently worked in a public health profession and were aged at least 18 years were eligible. Once theoretical saturation was reached, recruitment was stopped. There were no exclusion criteria related to sex, gender, race/ethnicity, or any sociodemographic or geographic characteristics, except for age of 18 years and working in the United States. Snowball sampling can be an effective tool [12], especially when a specific target audience cannot be reached through utilization of standard random sampling. 

To assess study population characteristics, participants were asked for their workplace setting, job title, types/groups of patient/clients they primarily served, if they provided any COVID-19-related services, as well as demographic characteristics. Additionally, we assessed participants’ psychological distress using the validated Kessler 6-scale [13], where a score of 13 or higher is marked as serious psychological distress. Poor sleep health was assessed using the two following questions: “*How often do you feel tired or fatigued after your sleep?*” and “*During your wake time, do you feel tired, fatigued, or not up to par?*”. We specifically assessed “*wake time*” instead of the usual “*daytime*”, as public health and related field professionals have night shifts, especially during critical times, such as a pandemic. 

In addition, we evaluated workplace needs and impact on life (“*Have providing COVID-19 related services impacted your personal life?*” and “*Do you feel you can benefit from a support group to discuss your experiences in providing COVID-19-related services?*”). 

Additional open-ended questions surveyed among participants addressed barriers faced when providing COVID-19 related services (“*What barriers, if any, have you faced when providing COVID-19-related services?*”); resources needed at the workplace to provide better services (“*What resources could be made available at your workplace in order to better provide COVID-19-related services?, In your expertise and experience, what additional information would you like to share regarding needed services for COVID-19 [for healthcare professionals and/or general population]?*”, and perceived and expressed needs of the general population related to COVID-19 services, as well as barriers to compliance (“*What are common barriers you feel that the general population may face when being COVID-19 policy compliant [such as self-isolation, use of masks, etc.]?”, “What type of questions, concerns, etc., if any, have you faced from your target audience regarding COVID-19 compliance [such as self-isolation, use of masks, etc.]?*”). 

To assess participants’ characteristics, including demographics and workplace, as well as prevalence of psychological distress, sleep health, perception of personal life being impacted, and need for a support system, we conducted descriptive statistics using SPSS (version 28, IBM).

Qualitative data were thematically analyzed. We first analyzed each individual open-ended question to identify emergent themes. During this phase, common words and phrases of each individual question were identified and then grouped together into common categories. These in turn were grouped into larger themes. The process was repeated until saturation was reached. Next, we used the same approach but across all open-ended questions to assess if there were any central emergent theme, independent of the question itself. Some supporting quotes from participants were edited for minor grammatical/spelling errors without changing the content, and any identifiable information regarding their place of work was excluded to ensure privacy as required by ethical approval guidelines. The study was approved by the Institutional Review Board.

## 3. Results

The recruitment was stopped at 22 participants, i.e., theoretical saturation. As shown in Table 1, a majority (58.8%) of the study participants were females and were aged 24–34 years (64.7%); there were no participants aged 55 years or higher. A higher percentage reported working for county or city public health departments, followed by non-profit organizations focused on health, with nearly 71% directly involved in providing COVID-19-related services. 

As shown in Table 2, 35.3% of the participants had psychological distress, with 23.5% of them having serious psychological distress. Another 88% reported feelings of tiredness and fatigue (both after sleep and during their wake time). When asked if support groups were needed to discuss work-related stressors, 47.2% reported yes while 35.3% reported unsure, and another large percentage (35.3%) did not answer the question. 

Furthermore, a majority (64.7%) of the participants reported they felt that their personal life was impacted by work related to providing COVID-19 services. Thematic analysis of qualitative responses further show that most participants were not only concerned about their own safety due to exposure to COVID-19, but also risking bringing the infectious disease home to their family, especially to young children. Likewise, some felt that their changing work schedule and increase in work hours cumulatively strained their time with family, reduced their ability to meet family needs, and added stress.

*“Having young children and a high-risk child at home does weigh on my personal life during this time. I can only take all the precautions and hope not to bring any possible infections home.”*

*“Time needed to adjust to changes have been much greater than anticipated taking away from meeting family needs.”*

*“Workload has increased, and average daily work time is approximately 12 h.”*

Next, we assessed the commonly cited workplace barriers that participants faced, with responses highlighting subthemes of workforce resources, public perception, and incongruent governance. For instance, participants noted limited resources, such as supplies and staff, made it difficult to provide timely COVID-19 related services, and, often, those in other departments had to be relocated, further leading to a negative impact on other needed services as well. Likewise, participants noted that lack of personal protective equipment, availability of timely training to address the changing nature of the pandemic, etc., all cumulatively limited effective service delivery. Further, participants noted that local political pushback and public perception against pandemic mitigation strategies, as well as a lack of consistent policies in such strategies, further served as substantial barriers to ensuring needed resources.

*“Frequent changing of information in the hospital. There wasn’t a centralized place to get updates information…”*

*“People believing everything they see or read on the internet and decide to call the call center to tell us we are lying to them.”*

*“Some staff in our program have been pulled for COVID-19 services, and unfortunately other services in our program, which is still running, may be delayed or not get done.”*

Further, adequate and centralized workforce resources, resources for the public, as well as funding to support public health services, were identified sub-themes mentioned by participants. For example, participants noted that having a plan of action is critical for outbreak case investigation, implementing steps to mitigate such an outbreak, as well as references for testing and results interpretation; these instructions all being centralized and easily accessible is imperative. Participants also reported that adequately trained staff, especially with respect to handling non-compliant clients and personal protective equipment, were needed resources. Many participants also acknowledged the disproportionate burden shared among the most vulnerable populations, including worsened mental health outcomes, and the need for age-appropriate and culturally appropriate health information related to COVID-19 prevention. Cumulatively, participants reported such needed resources were only feasible if appropriate funding was allocated to public health infrastructure.

*“Better communication about how decisions are made.”*

*“Up-to-date information and research on relevant topics.”*

*“At first would be the systematic process of having the drive up testing centers, enough test kits to test patients.”*

*“During a pandemic it would be nice if everyone was on the same page.”*

*“It [pandemic] magnified health disparities marginalized communities face (lack of phones, poor WiFi service, lack of computer to do telehealth, health literacy…).”*

Participants were also asked the common types of questions, needs, and concerns they saw the general population express, as well as their perception of barriers that the general population faced to ensure COVID-19 compliance. Responses highlight the lack of valid, adequate, and consistent health information, safety concerns, as well as emotional and financial burden among the general population as recurring sub-themes. For instance, participants reported that there was no centralized source of information for the general population, and often conflicting evidence was presented from a variety of sources, including governmental sources and employers on compliance policies, which in turn led the population to disbelieve information. Participants also reported that not all information was up to date and there remains a need to provide information on resources for testing, treatment, healthcare services, etc.

*“…community members not adhering to county protocols, employers not implementing safety measures in their establishments, and questions regarding covid19 transmission and exposure.”*

*“Inadequate or poor understanding of rationale behind face covering, inappropriate use of gloves in public, denial of scientific evidence regarding self-isolation and infection control.”*

Cumulatively, the lack of consistency in compliance from others and lack of reliable information have further led to fear of safety among the client population regarding COVID-19, including routine healthcare utilization.

*“How their [clients’] chronic condition will [be] impacted if they contract COVID-19; continuity of care; impact of not receiving care specific to their chronic condition.”*

*“Concerned about being around others, being separated from their babies in the hospital, and their support persons not allowed in the delivery room/hospital when they give birth.”*

*“Attending medical appointments, prenatal check ups, yearly well-child appointments due to medical offices being closed or fear of contracting virus.”*

*“Coming to the ER. People are afraid to come [to] the ER for legitimate reasons because they’re more afraid of getting sick.”*

Participants also reported that many of their clients suffered both poor mental health and financial burden due to the pandemic, with disproportionate burden noted among the most vulnerable.

*“Isolation can wear on people and effect their mental state. Also, with now the option to use mask in some cities those of the higher risk category are even more fearful of leaving their homes.”*

Cumulatively, such qualitative assessment highlighted three core themes: (1) workforce capital (staff, training, funding, and centralization of timely information) in order to provide public health service (including health education, COVID-19 mitigation, etc.), (2) health literacy capacity building for the general population to make informed decisions, and (3) need for legislative consistency. 

For example, results show that a consistent theme across all responses was the need for workforce resources. This included staffing, timely staff training to address emergent public health needs, timely and adequate scientific information to provide services and answer questions from clients, as well as training on handling non-compliance.

Likewise, across all responses, another consistent theme was that of health literacy capacity building for the general population. Multiple conflicting sources of information, lack of adequate resources to vet such information, and lack of a centralized source were all cited as reasons leading to distrust of public health professionals from the public. 

Finally, lack of consistent policy among public health workforces on COVID-19 mitigation strategies, politicization of such strategies, lack of consistent policies on masks and social distancing across local jurisdictions, as well as lack of enforcement all cumulatively demonstrate a need for cohesive policies from legislators and decision-makers.

## 4. Discussion

The COVID-19 pandemic has impacted nearly all sectors of daily life, with a disproportionate burden among frontline workers, including the public health workforce (PHW). While current empirical evidence highlights the mental and physical burden of healthcare professionals, few have addressed such experiences among the PHW, who are often tasked with outbreak investigation, prevention, as well as compliance regulations. In this study, we aimed to assess the commonly faced barriers and needed resources for the PHW in order to address the current barriers faced as well as expectations for needed resources to provide effective services for future public health emergencies. 

Both qualitative and quantitative results of our study demonstrate that the PHW experienced poor mental health and sleep health, as well as strained family dynamics resulting from the pandemic. This is consistent with the previous literature on other healthcare workers. In a study among mental healthcare providers, [14] noted that participants reported fatigue from providing teletherapy, dissatisfaction with their work and its meaningfulness, as well as negative impacts on personal life. Likewise, Bryant-Genevier and colleagues [15] found over 30% prevalence of depression, anxiety, and PTSD among their participants who were in the PHW. In China, a similar study among PHW members found that workload, as assessed through effort and over-commitment, were related to negative mental health outcomes [16]. Our results further add to the literature, noting that, often, concerns of safety for family, especially chronically ill family members, heightened the stressors experienced by the PHW, as did the extended work hours with limited resources. Further, a recent assessment of global expert opinion by [17] demonstrated the critical gap in research into workforce development needs. Our study helps address this gap by providing direct feedback from PHW staff on the need for funding, staff, and continued training to adequately address emergent public health needs. 

Another unique outcome noted in our results was that, despite high prevalence of markers for distress, majority of the participants did not want to address the need for support systems for themselves. This is consistent with the previous literature related to other professionals that highlight low help-seeking behavior among the most in need. For example, a study among Indian healthcare workers during the COVID-19 pandemic demonstrated that, despite presence of anxiety and other stressors, only 17% reported they wanted psychological help [18]. A similar pattern was noted in China, where less than 13% of the PHW sought mental health support [19]. Our study in the United States further highlights this global phenomenon and, thus, the need for workplace wellness programs, especially those promoting help-seeking behavior among PHW staff. 

In addition, community health literacy on the pandemic and mitigation strategies was another major barrier identified by our study participants. Low health literacy has been associated with low cancer screening behavior [20], risky health behaviors [21], and even COVID-19 vaccine hesitancy [22]. Our study, along with the existing literature on other health disparities, thus further highlights the need for community-based efforts to improve health literacy, including understanding medical information, navigating the wealth of information to decipher the accurate content, as well as capacity for the public to make informed decisions regarding their own health. 

Finally, our study highlights that the politicization of the pandemic and its mitigation strategy has further served as a barrier for the PHW to provide adequate care. The literature further highlights that the impact of politics on COVID-19 response has substantially impacted vaccine acceptance and enabled spread of misinformation on management strategies [21,23,24]. As further shown in our study, consistent science-based information from legislators and consistency in policy regulation are of imperative need. 

The results of this study should be interpreted in the context of its limitations and strengths. Given the cross-sectional study method of analyzing data at a single point in time, it is difficult to draw causal conclusions of long-term mental health outcomes of PHW. Another limitation is that there is a risk of self-selection bias, given no incentive was offered for study participation. Additionally, there is a risk of social desirability bias, particularly for sensitive questions. Participants may choose to under-report or not report negative views, emotions, or behaviors that may be perceived as undesirable attributes. Both self-selection bias and social desirability bias may influence the validity of the study. 

Notwithstanding such limitations, a major strength of this study is the mixed-methodology that enables us to explore the unique experiences and needs of the PHW. As the survey instrument contained open-ended questions and participants were permitted to respond anonymously, responses provided contextual data to explore further and establish central themes. Additionally, quantitative measures were integrated to supplement the findings and, thus, provide a comprehensive assessment. Further, the majority of the current studies on workforce fatigue focus on healthcare staff, who are often considered frontline. Nevertheless, members of the PHW are also frontline, as they not only conduct contact tracing, but also are directly involved in preventive measures. Our results also highlight the need for more concrete workplace policies, such as routine training on surveillance mechanisms (including through continuing education credits), putative development of pipeline programs to train graduate students to enter the public health workforce with skills needed to address the next emergent public health need, as well as legislative action through funds to promote health literacy in the community to empower the general population to discern facts from fiction and make informed health decisions. 

## 5. Conclusions

Our study demonstrated an overarching theme of the need for paradigm shift in capacity building for public health emergency response. There remains an imperative need for both micro- and macro-level resources, such as staffing, continued training, infrastructure, and community and political support. The results of this study highlight the importance of investment in public health infrastructure to improve emergency response without leading to workforce fatigue, through comprehensive workplace health promotion policies. 

## Figures and Tables

**Table 1:** Study population characteristics.

**Sex**	
Female	58.8%
Male	35.3%
Other	5.9%
**Age group**	
25–34 years	64.7%
35–54 years	35.3%
**Primary site of employment**	
County/City Public Health Department only	35.3%
Hospital/Clinic only	11.8%
Non-profit Organization focused on health only	29.4%
Academia	11.8%
More than one	11.8%

**Table 2:** Study population’s mental and sleep health outcomes.

Psychological Distress	
None	64.7%
Mild-to-moderate	11.8%
Serious	23.5%
Individual psychological distress variable. During the past 30 days, frequent feeling of being…	
…nervous	64.7%
…hopeless	29.4%
…restless or fidgety	70.6%
…so depressed that nothing could cheer you up	29.4%
…that everything was an effort	47.1%
…worthless	23.5%
Sleep health	
Frequent feeling of tired of fatigue after sleep	88.2%
Frequent feeling of tired, fatigued, or not up to par during wake time	88.2%

## Data Availability

No public access data are available, per guidelines of ethical approval.
